# Book Reviews

**Published:** 1872-03-01

**Authors:** 


### Book Reviews.

First Biennial Report of the State Board of Health of California, for the years 1870 and 1871. Sacramento, Cal., 1871.

This is a volume, in paper cover, of 203 pages, 90 of which is occupied by the report of the Board proper, and 113 by various papers under the head of Appendix. The California State Board of Health was created by a special act of the Legislature, for the purpose of promoting the vital and sanitary interests of the State. The present members of the Board are Henry Gibbons, Sr., M. D., of San Francisco, President; Thomas M. Logan, M. D., of Sacramento, permanent Secretary; J. F. Montgomery, M. D., of Sacramento; L. C. Lane, M. D., of San Francisco; F. Walton Todd, M. D., of Stockton; C. E. Stone, M. D., of Marysville, and Luke Robinson, M. D., of Colusa. The present report embraces a great variety of topics of much interest to all who desire reliable information concerning the sanitary condition and advantages of the Pacific side of the great mountain region. The question where to send invalids for a change of climate, whether consumptives or otherwise, is of importance to every practising physician. As bearing on this subject, we quote the following from the report of M. H. Biggs, M. D., in reference to Santa Barbara :

"I beg to make a few remarks in regard to the climate of that portion of the county of Santa Barbara which consists of a narrow strip of land lying on the coast, beginning at Point Conception and ending at Point San Buenaventura, a distance of about sixty miles from west to east, bounded on the north by a high range of mountains called the Coast Range, and on the south by the ocean. The width of this strip is from two to four miles. About two-thirds of it is undulating or rolling land, the rest is in level spots, and all of the richest soil.

The principal feature of this region consists in, first, the almost due easterly and westerly direction of its coast ; secondly, the unbroken range of mountains, about three thousand feet high, which form a perfect barrier to and protection from the severe north and northeast winds which are so annoying and detrimental to health elsewhere ; and thirdly, in a chain of islands which lies along this favored portion of the coast, to protect it from the cold ocean fogs that roll in so uninterruptedly in other parts. These three peculiarities combined give that exceptional climate with which Santa Barbara is blessed ; its equal not being found on the Pacific coast, at least not outside of the tropics. The channel of Santa Barbara lies between these islands and the main land, and extends all the way from Point Conception to San Buenaventura. Every one, upon entering it, in coming down the coast especially, remarks the instant change of temperature after rounding the Point, and sometimes suddenly emerging from a thick driving fog and finding himself under a bright blue sky, with warm, pleasant weather, and to make the transition more complete, feels himself gliding along in smooth water instead of plunging through a heavy sea. Five minutes after passing the light-house on the Point, all the wretched sea-sick passengers on the steamer crawl out of their berths and go on deck to enjoy the delightful change.

"Our prevailing winds are west and northwesterly during the summer or dry months, and southeasterly during the winter or wet months. The wet season extends from about the middle of December to the end of March, with a rain fall of from ten to eighteen or twenty inches. The average temperature of summer is below seventy degrees, and about fifty-three degrees during winter. Frost is rarely seen, and sudden great changes in temperature never felt. It is considered exceedingly warm should the thermometer rise to seventy-eight or eighty degrees at noon, and it hardly ever exceeds this degree unless caused by fires in the neighboring mountains, which now and then occur during the fall of the year, when everything is dry.

"The most remarkable fact in regard to this region is the seeming impossibility for epidemics to visit it. The small-pox has ravaged the whole country three or four times since 1843. It has been singularly virulent in San Luis Obispo, the first town north of us, and also in Los Angeles, which lies in the next county south. During the prevalence of the epidemic, persons have come from these neighboring towns with the seeds of the disease, and have died in Santa Barbara within a few days of arriving, but some antiseptic property in the climate has prevented the contagion, and it has never spread. Scarlatina and diphtheria are unknown here. There are some instances of the measles having been brought here, and perhaps run in a very mild form through some of the members of the family first bringing the disease, but it has never spread as an epidemic. There are no malarious fevers. Persons who came here afflicted with fever and ague, rarely have more than two or three attacks. They soon become well, often even, without the use of antiperiodics. The climate seems sufficient to cure the malady. During a residence of twenty years I have only seen one case of membranous croup, and heard of two others. There is no disease endemic to Santa Barbara —nothing but what can usually be referred, either directly or indirectly, to some indiscretion in eating or drinking, or from unreasonable exposure.

"Many consumptives visit this part of the country, and derive much benefit from its equable climate, which makes it especially appropriate to this class of patients, who besides find much attraction in the pleasant drives along the hard sea beach, or in the healthful rides and walks up the many romantic little dells and valleys, and over the low rolling hills, covered here and there with clumps of evergreen, oak, sycamore, cottonwood, laurel, alder, and other trees and shrubs too numerous to mention. Game is likewise plentiful. We have good fishing in the mountain streams and in amongst the kelp in the channel, and every other attraction that one of these days will make Santa Barbara the favorite resort of invalids, convalescents, and all those who desire to pass a quiet, peaceful and healthy life.

"I bring this slight sketch of Santa Barbara before the State Medical Association, suggestively, in case the question should ever be mooted as to the most proper place for the establishment of a State Hospital or Sanatarium."

Earth as a Topical Application in Surgery. Being a full exposition of its use in all the cases requiring topical applications, admitted into the Men's and Women's Surgical Wards of the Pennsylvania Hospital, during a period of six months, in 1859. By Addinell Hewson, M.D., one of the attending surgeons of the Pennsylvania Hospital. With four photo-relief illustrations. Philadelphia: Lindsay & Blakiston. 1872.

This is a neatly published volume of 309 pages, octavo; and contains a full explanation of the mode of using earth as a surgical dressing, with a large number of cases illustrating its advantages; and a closing chapter on the *modus operandi* of the remedy. The subject is one of much practical importance, and the book will amply repay both physician and surgeon for the time involved in its careful reading. For sale by S. C. Griggs & Co., Chicago. Price $2.50.

A Practical Treatise on the Diseases of Woman. By T. Gaillard Thomas, M.D., Prof. of Obstetrics and Diseases of Women and Children, in the College of Physicians and Surgeons, New York; Physician to the Rosevelt Hospital, New York, etc., etc., etc. Third edition, enlarged and thoroughly revised, with two hundred and forty-six illustrations on wood. Philadelphia: Henry C. Lea. 1872. Octavo, pp. 784.

The former editions of this work are so well known to the profession that little need be said concerning its general merits. The present edition shows a careful revision, and the addition of sufficient new matter to increase its size twenty-five per cent. It is written and published in good style, well illustrated, and constitutes one of the best practical works accessible to the student and practitioner. Our copy was received through the house of S. C. Griggs & Co., Chicago. Price $6.00.

Transactions of the Medical Society of the State of California. Years 1870–71. Sacramento, Cal. 1872.

This is a volume of 253 pages, published in good style, in paper cover, and contains the record of proceedings of the Society, and the addresses and papers read before the same. Among the more important papers are a Report on Surgery, by G. L. Simmons, M.D. of Sacramento; Coxalgia and its treatment, by L. C. Lane, M.D., of San Francisco; Medical Topography of San Diego, by D. B. Hoffman, M.D.. of San Diego; Medical Topography of Santa Barbara, by M. H. Biggs, M.D., of Santa Barbara; On Quarantine, by W. I. Wythe, M.D., of Sacramento; Medical Topography of Petaluma, by G. W. Graves, M. D., of Petaluma; Why do we wear spectacles? by Ed. M. Curtis, M.D., of Sacramento; Ophthalmia and Aural Surgery, by W. F. Smith, M.D., of San Francisco; Hygiene, as regards the sewerage of San Francisco, by Arthur B. Stout, M.D., of San Francisco; Report of Committee on Practical Medicine, by Henry Gibbons, Sr., M.D., of San Francisco; Monthly and Annual Mortality of California, and the Corresponding Mean Temperature and Humidity at Sacramento, by Thos. M. Logan, M.D., of Sacramento. Everything about this volume goes to prove the existence of an active and valuable medical organization in the young State of California.

Transactions of Iowa State Medical Society.

This is the first volume of transactions published by the Iowa State Medical Society, and is a very creditable production. It includes the proceedings of the society for the years 1867–68–69–70, and a selection of the papers presented at each of the meetings.

Thyrotomy for the removal of Laryngial Growths, modified by Ephraim Cutter, M. D., Woburn, Mass. A Monograph.

Electricity in the Treatment of Diseases of the Skin, by George M. Beard, M. D.

Transactions of the Wisconsin State Medical Society, 1871.

A handsome volume containing a number of valuable and interesting reports.

Transactions of the Medical Society of the State of West Virginia, 1871.

A case of Hydrocele of the Round Ligament mistaken for, and operated upon, as a strangulated Hernia, with remarks, by Charles A. Hart, M. D. New York: D. Appleton & Co.

